# Foreword: Lesson learning about getting research into policy and practice

**DOI:** 10.1186/1478-4505-9-S1-S1

**Published:** 2011-06-16

**Authors:** C Whitty, S Kinn

**Affiliations:** 1 Director Research and Evidence Division, Department for International Development, 1 Palace Street, London SW1E 5HE; 2 Head of Health Research, Department for International Development, Abercrombie House, Eaglesham Road, East Kilbride, Glasgow G75 8EA

## Abstract

**Foreword:**

The UK Department for International Development (DFID) is committed to investing in research to combat poverty, reduce high mortality and morbidity in resource poor contexts and support progress towards meeting the Millennium Development Goals. Research helps us to identify what works, what does not work and how to understand the local context when introducing new ways of working. There is no point doing research if the findings do not get into policy and practice. DFID strongly encourages all research programmes to consider research uptake activities as an integral part of the research.

This special supplement draws on the work of the Sexual Health HIV Evidence into Practice (SHHEP) initiative. SHHEP is a collaboration across four DFID Research Programme Consortia (RPC) that undertake research and action on HIV and Sexual and Reproductive Health in resource poor contexts. Each consortium consists of 5 or more research, advocacy or service provider institutions from the south and the north working together over a five year period on critical areas of sexual and reproductive health. The essence of SHHEP is to share learning on research uptake and research engagement in Sexual and Reproductive Health, including HIV. The group has formulated a range of targeted mechanisms to communicate health research to different audiences and spearhead change, and were finalists for the British Medical Journal 2010 Getting Research into Practice (GRiP) prize.

The papers in this special supplement focus on lesson learning on getting research into policy and practice. They highlight the range of methodologies and approaches researchers and communication specialists have used in different contexts to try to ensure research does not simply gather dust on library shelves but feeds into and is relevant to policy and practice in different contexts (for example South Africa, Swaziland, Tanzania, Uganda, Malawi, Ghana, Bangladesh) and on a diversity of topic areas (Gender based violence, sexualities, orphans and vulnerable children, HIV care and treatment including male circumcision, cotrimoxazole and links with nutrition).

The work reported in this supplement provides examples of approaches that have been tried and from which other researchers can learn. They demonstrate that getting research into policy and practice is complex, dynamic and multi-faceted; and a wide range of context and issue specific conceptual and practical approaches have to be used. I hope that the innovative approaches and promising ways forward, presented in these papers, will inspire and motivate others.

Professor Christopher Whitty

Director Research and Evidence Division

Department for International Development

Dr Sue Kinn

Head of Health Research

Department for International Development

## Competing interests

The authors declare that they have no competing interests.

